# Syncope and New-Onset Bifascicular Block Associated With Intravenous Immunoglobulin in a Patient With Chronic Inflammatory Demyelinating Polyneuropathy

**DOI:** 10.7759/cureus.82010

**Published:** 2025-04-10

**Authors:** Trevor Wolchover, Harpreet Dosanjh, Jonathan Lalezari, Isaac Delote, Jasprit Takher

**Affiliations:** 1 Internal Medicine, Los Robles Regional Medical Center, Thousand Oaks, USA

**Keywords:** bifascicular block, bradycardia, cardiology, chronic inflammatory demyelinating polyneuropathy, clinical medicine, electrocardiogram (ecg/ekg), intravenous immunoglobulins (ivig), side effects, syncope

## Abstract

Chronic inflammatory demyelinating polyneuropathy (CIDP) is an immune-mediated neuropathy often treated with intravenous immunoglobulin (IVIG), which is generally well-tolerated but has occasionally been associated with rare cardiovascular side effects. We present the case of an 84-year-old male with CIDP, maintained on biweekly IVIG infusions, who experienced a syncopal episode with documented bradycardia of 30 beats per minute (bpm) during an infusion. Upon evaluation in the emergency department, the patient was found to have a new bifascicular block requiring pacemaker placement. This case underscores a rare adverse reaction associated with IVIG infusion, highlighting the importance of careful cardiac monitoring in older patients with CIDP and underlying or potential cardiac risk factors.

## Introduction

Chronic inflammatory demyelinating polyneuropathy (CIDP) is an autoimmune disorder characterized by progressive demyelination of peripheral nerves, leading to motor and sensory deficits. Treatment options primarily include corticosteroids, plasma exchange, and intravenous immunoglobulin (IVIG), the latter being highly effective in stabilizing disease progression and reducing symptoms for many patients [1].

Although IVIG is generally well-tolerated, serious adverse effects have been reported, including thromboembolic events, myocardial infarction, and arrhythmias, although these remain uncommon [2]. The incidence of IVIG-associated cardiovascular side effects is estimated to be 0.6-1.2% in patients receiving treatment [3,4], with bradycardia and conduction disturbances occurring in an even smaller fraction of cases. Risk factors include older age, preexisting cardiac disease, and underlying inflammatory states that may exacerbate endothelial dysfunction [5].

Cardiac side effects from IVIG are believed to arise due to increased blood viscosity, transient inflammatory cytokine release, and immune-mediated effects on the myocardium [6]. These adverse reactions typically present as transient bradycardia, conduction delays, or, in rare cases, more severe arrhythmias necessitating intervention [7,8]. This report describes an elderly male with CIDP who developed a new bifascicular block following IVIG infusion, emphasizing the need for vigilance in cardiovascular monitoring for high-risk populations.

## Case presentation

An 84-year-old male with a history of CIDP and hypertension presented to the emergency department after experiencing a syncopal episode during his routine IVIG infusion. He had been receiving biweekly IVIG infusions for CIDP management for the past 10 years and had previously tolerated the treatment well without significant adverse effects.

During his latest IVIG infusion, approximately 20 minutes into administration, the patient developed a sudden onset of lightheadedness, diaphoresis, and shortness of breath, followed by a brief loss of consciousness lasting approximately 30 seconds. At the infusion center, he was pale and unresponsive, prompting immediate discontinuation of IVIG and activation of emergency medical services. Telemetry monitoring at the infusion center recorded a heart rate of 30 bpm during the syncopal episode, concerning for significant bradycardia. The patient spontaneously regained consciousness without postictal confusion, seizure activity, or focal neurological deficits. He was subsequently transported to the emergency department (ED) for further evaluation.

Upon arrival at the ED, the patient was alert but reported persistent fatigue and mild dizziness. His vital signs on admission were as follows: heart rate 68 bpm, respiratory rate 19 breaths per minute, blood pressure 131/81 mmHg, and oxygen saturation 94% on room air. On physical examination, he was neurologically intact beyond his baseline CIDP-related deficits, and there were no new focal neurological findings. Cardiovascular examination revealed a regular bradycardic rhythm without murmurs, rubs, or gallops, and no signs of heart failure or volume overload were present. Given his recent IVIG infusion, transient bradycardia, and unexplained syncopal episode, an extensive workup was initiated.

Laboratory investigations revealed mild renal impairment, with a creatinine level of 1.34 mg/dL. However, serum sodium, potassium, glucose, and calcium were within normal limits, and cardiac biomarkers, including troponin I and B-natriuretic peptide, were negative, ruling out an acute coronary syndrome (Table 1).

**Table 1:** Laboratory results on admission notable for mild renal dysfunction but were otherwise unremarkable for acute metabolic derangements or electrolyte imbalances

Laboratory test	Result	Reference range
Sodium (Na)	136 mmol/L	136-145 mmol/L
Potassium (K)	4.7 mmol/L	3.6-5.1 mmol/L
Chloride (Cl)	107 mmol/L	98-107 mmol/L
Creatinine	1.34 mg/dL	0.6-1.2 mg/dL
Blood urea nitrogen (BUN)	24 mg/dL	7-18 mg/dL
Glucose	101 mg/dL	77-99 mg/dL
Calcium (Ca)	9.2 mg/dL	8.5-10.1 mg/dL
Troponin-I high sensitivity	14 ng/L	0-78 ng/L
B-natriuretic peptide (BNP)	22 pg/mL	0-99 pg/mL

**Figure 1:**
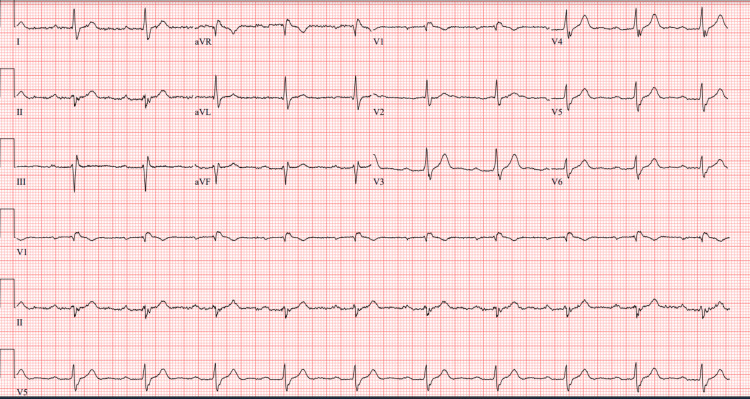
ECG on admission showing sinus rhythm with a first-degree atrioventricular (AV) block, a right bundle branch block (RBBB) with left anterior fascicular block (LAFB), manifested as a left-axis deviation, consistent with a new bifascicular block. Heart rate (HR) = 61, PR interval = 294 ms, QRS = 130 ms, QT/QTcB = 404/406 ms

**Figure 2:**
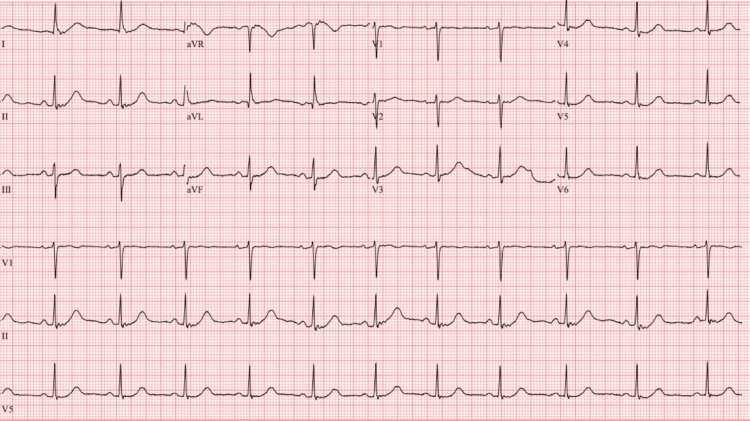
ECG six months prior demonstrating normal sinus rhythm. Heart rate (HR) = 68 bpm, PR interval = 166 ms, QRS = 76 ms, QT/QTcB = 434/461 ms

A 12-lead electrocardiogram (ECG) obtained upon admission revealed sinus rhythm with a prolonged PR interval (first-degree AV block), a right bundle branch block (RBBB) with left anterior fascicular block (LAFB), manifested as left axis deviation, consistent with a new bifascicular block (Figure 1). A previous ECG obtained six months prior had demonstrated normal sinus rhythm (Figure 2), confirming that this conduction abnormality was newly acquired.

Given the close temporal relationship between IVIG administration and the onset of cardiac conduction abnormalities, a potential causal association was considered. The patient was determined to be at high risk for progression to complete heart block, and after discussing risks and benefits, the decision was made to proceed with permanent pacemaker implantation to prevent further bradycardic episodes.

The patient subsequently underwent an uneventful dual-chamber pacemaker placement, with resolution of symptoms post-procedure. Telemetry monitoring following pacemaker implantation showed a stable heart rate without further conduction abnormalities. Over the next 48 hours, he remained clinically stable, was ambulatory without further syncopal episodes, and was subsequently discharged home in stable condition.

## Discussion

This case illustrates a rare but significant cardiovascular complication associated with IVIG therapy in a patient with CIDP. While IVIG remains a mainstay in CIDP treatment, its potential to cause cardiac conduction abnormalities, including bradycardia and heart block, remains underrecognized. Although IVIG-related cardiac events are generally uncommon, with an estimated incidence between 0.6% and 1.2% [3,4], cases involving conduction disturbances severe enough to require pacemaker implantation are exceptionally rare. A literature review suggests that fewer than 15 case reports have documented patients experiencing IVIG-induced bradycardia or conduction blocks necessitating pacemaker placement [4,7]. Given this low incidence, IVIG-associated arrhythmias may be underreported or overlooked, particularly in patients with preexisting cardiac risk factors.

The exact mechanisms underlying IVIG-induced conduction abnormalities are not fully understood, but several theories have been proposed. One potential explanation is the transient increase in blood viscosity following IVIG administration, which could impair myocardial perfusion and contribute to conduction delays [5]. In addition, IVIG is known to induce cytokine release, triggering inflammatory responses that may transiently disrupt the autonomic regulation of cardiac rhythm [6]. Some studies suggest that IVIG may have direct immune-mediated effects on ion channels within the cardiac conduction system, leading to bradyarrhythmias or conduction blocks [4]. Regardless of the precise mechanism, evidence from prior case reports indicates that IVIG-associated arrhythmias can occur across a wide demographic spectrum, including both pediatric and adult patients, highlighting the need for increased awareness among clinicians [5,6].

Several reports have described similar presentations, supporting a temporal association between IVIG administration and cardiac conduction abnormalities. One case report documented a patient who developed profound bradycardia following IVIG infusion in idiopathic thrombocytopenic purpura [7]. Another described cardiac rhythm abnormalities occurring during IVIG therapy in two newborn infants, reinforcing that this phenomenon is not strictly age-dependent [6]. In addition, a 2024 case report documented a high-degree atrioventricular block in a patient receiving IVIG, further supporting the link between IVIG and conduction disturbances [4]. These cases, along with the present one, highlight the potential for IVIG to provoke clinically significant bradyarrhythmias, even in patients without a prior history of conduction disturbances.

This case reinforces the importance of careful cardiac monitoring in high-risk patients receiving IVIG therapy, particularly those who are older or have underlying cardiovascular disease. Given the unpredictability of IVIG-related conduction abnormalities, clinicians should consider routine ECG monitoring before and after infusion, as well as increased vigilance for symptoms such as lightheadedness, palpitations, or syncope. Future research is needed to better characterize the pathophysiology of IVIG-induced conduction disturbances and to establish standardized monitoring guidelines that may help prevent adverse cardiovascular outcomes in vulnerable populations [3,4].

## Conclusions

This case highlights the potential for IVIG therapy to provoke conduction disturbances in susceptible patients, particularly those with advanced age or underlying cardiovascular risk factors. In this case, an elderly male with CIDP receiving biweekly IVIG developed a new bifascicular block, necessitating pacemaker placement following a syncopal episode.

Clinicians should consider cardiac monitoring for CIDP patients receiving IVIG therapy, especially those with any history of conduction abnormalities or symptoms suggestive of arrhythmia. Further research is needed to better understand the association between IVIG therapy and cardiovascular complications, guiding monitoring protocols to mitigate risk in vulnerable populations.
